# Assessment of testicular parenchyma in COVID-19 patients: a case–control study using ultrasound histogram analysis

**DOI:** 10.1590/1806-9282.20250074

**Published:** 2025-06-16

**Authors:** Mehmet Demir, Hatem Kazımoğlu

**Affiliations:** 1 Harran University, Faculty of Medicine, Department of Radiology – Şanlıurfa, Turkey.; 2 Sanko University Hospital, Faculty of Medicine, Department of Urology – Gaziantep, Turkey,

**Keywords:** Ultrasonography, Image interpretation, Computer-assisted, Infertility, COVID-19

## Abstract

**OBJECTIVE:**

Uncontrolled activation of immune cells, such as lymphocytes and macrophages, can result in heightened inflammatory cytokine production, leading to oxidative stress and cell death in various organs, including the brain and testes. The systemic effects of COVID-19 are not limited to the respiratory system and can also affect other organs. The aim of this study was to focus on the possible effects of COVID-19 infection on the testicles in male patients.

**METHODS:**

Ultrasound images of 50 patients who were shown to have COVID-19 infection by polymerase chain reaction method and 50 healthy volunteers who did not have any complaints and were polymerase chain reaction negative were evaluated retrospectively. While forming the patient and control groups, people who smoked or had diseases such as varicocele, which caused testicular parenchyma to be affected, were excluded from the study. Tissue histogram analysis was performed on the images obtained, including the entire testicular parenchyma, and radiomics were obtained. The Mann-Whitney U test and independent samples t-test were used to compare parametric and non-parametric results in independent groups.

**RESULTS:**

The average age of the patient group and control group was 32.13±7.3 years and 31.23±7.9 years, respectively, and no statistically significant difference was detected (p>0.05). When testicular volumes in the patient and control groups were compared, no statistically significant difference was detected (p=0.58). Statistically significant differences were found between the two groups in the radiomics examined using skewness of histogram, zone-size non-uniformity of gray-level size-zone matrix, information measure of correlation of gray-level co-occurrence matrix, run percentage of gray-level run-length matrix, high gray-level run emphasis of gray-level run-length matrix, and kurtosis of histogram (p<0.05). There was no significant difference in other parameters.

**CONCLUSION:**

Ultrasound histogram analysis may be useful in showing heterogeneity, which is an indicator of the impact on the testicular parenchyma in people with COVID-19 infection. Thus, in addition to other clinical findings and laboratory parameters, ultrasound histogram analysis may be helpful in demonstrating testicular parenchymal involvement in the follow-up of patients after COVID-19.

## INTRODUCTION

The COVID-19 pandemic has been a major public health problem worldwide. The most common symptoms of the disease are cough, fever, shortness of breath, and respiratory symptoms. However, the systemic effects of COVID-19 are not limited to the respiratory system and can also affect other organs. This study will focus on the possible effects of COVID-19 infection on the testicles in male patients. Some recent studies have pointed to findings that may suggest that COVID-19 may have adverse effects on the testicles in men. Excessive activation of the immune system during a viral infection can lead to increased levels of pro-inflammatory substances, including interferon-gamma (INF-γ), tumor necrosis factor (TNF)-α, transforming growth factor (TGF)-β, and various interleukins (ILs)^1,2^. Elevated C-reactive protein (CRP) levels are commonly observed in clinical settings as indicative of viral infections^3^. Specifically, uncontrolled activation of immune cells, such as lymphocytes and macrophages, can result in heightened inflammatory cytokine production, leading to oxidative stress and cell death in various organs, including the brain and testes^4–6^. The testes, in particular, are highly susceptible to increased levels of inflammatory molecules and oxidative stress, with pro-inflammatory cytokines and oxidative stress adversely affecting steroidogenesis and spermatogenesis^4,5^. Under various pathological conditions, the abnormal release of cytokines, including elevated CRP and INF-γ, has been linked to disruptions in steroidogenesis and spermatogenesis in the testes. In light of the reported cytokine storm in COVID-19 cases, the testes face a heightened risk of structural and functional abnormalities, irrespective of direct SARS-CoV-2 infection^7,8^.

Testicular ultrasound is a non-invasive imaging method used to obtain images of the testicles and surrounding tissues. We may not be able to see the pixel-based differences in the obtained ultrasound images through our eyes. By converting these details in the image into numerical values as a result of computer-aided texture analysis, data called radiomics are obtained. By analyzing the overall image of the lesion, ultrasound radiomics can visualize the heterogeneity of the tissue^9^. Ultrasound radiomics, a machine learning method, analyzes the features of the image and creates a prediction model to increase the accuracy of early diagnosis. This study aimed to examine the testicular ultrasound images of male patients with COVID-19 infection by texture analysis.

## METHODS

### Population

This retrospective investigation adhered strictly to the ethical principles outlined in the Declaration of Helsinki and received the green light from the Harran University ethics committee, under the approval number 23.16.07. Group 1, comprising 50 patients (equating to 100 testicles), constituted of individuals who had triumphed over COVID-19 infection. After selecting 50 individuals who met the patient group requirement, people who volunteered to participate in the study and who had not had COVID-19 disease before were included randomly. Our study focused on capturing the imagery of male subjects aged 18 years and above, with confirmed COVID-19 diagnoses through reverse transcription polymerase chain reaction (RT-PCR) testing and recovery periods spanning between 4 and 12 weeks. The control group, untouched by the specter of COVID-19, comprised exclusively of hale and hearty individuals aged 18 years and above who had never received a COVID-19 diagnosis and produced negative RT-PCR results.

Criteria such as not smoking, not taking medication, and not having any other systemic or genital disease such as varicocele were taken into consideration in the members of the patient and control groups included in the study. It was tried to form a control group so that the average age of the members of the patient and control group would be similar. The members of the control group were volunteers who did not have COVID-19 disease or suspicion and who agreed to participate in the study.

It is worth noting that certain studies have illuminated the impact of conditions such as tumors, testicular torsion, orchitis, hydrocele, undescended testicles, or prior surgeries on the homogeneity of testicular parenchyma^10,11^. Consequently, our study consciously excluded individuals afflicted by these ailments. Furthermore, participants with known varicocele or any pre-existing testicular pathologies were meticulously omitted from the study.

### Image acquisition

**Figure 1:**
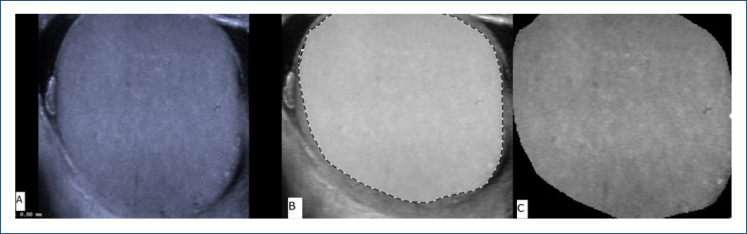
Processing of the testicular ultrasound image of a 30-year-old patient from the patient group: **(A)** sagittal image of the testicle in B-mode, **(B)** determination of the boundaries of the testicular parenchyma by drawing, **(C)** extraction of the image from the parenchyma with drawn borders and processing it in the MATLAB program.

In our study, ultrasound images of patients with COVID-19 who met the criteria that we kept in our hospital archive were used. Ultrasound images of the control group were prospectively recorded. Ultrasound images of the patient and control group were performed by M.D. who has 10 years of ultrasonography experience, armed with the mighty Siemens Acuson S2000, a high-frequency (4–14 MHz) linear array transducer known as the 9L4. The testicular parenchyma was marked in the sagittal section images, and thus, the region of interest (ROI) was extracted (Figure 1).

### Image analysis

The histogram analysis of the images was performed by M.B. who has 15 years of radiology experience and has been performing histogram analysis for 5 years. M.B. was blinded to the patient and control groups during the histogram analysis. Placement of the ROI for the histogram analysis was made by magnifying the image as much as possible, and then manually drawing from the borders which could be most clearly selected at the largest size. The histogram analysis was performed on the XML files using MATLAB v. 2009b software (MATrix LABoratory, Mathworks Inc., Natick, USA) (Figure 1). By evaluating the ROI, various calculations were made to analyze parameters, such as gray-level intensity and standard deviation of the histogram, entropy, uniformity, skewness, and kurtosis values. These calculations were carried out with respect to two specific size categories: size L% (representing mean low values from the area below the standard deviation) and size M% (representing the mean area remaining below the standard deviation).

### Statistical analysis

The data obtained from the statistical study were analyzed statistically using SPSS vr.25 software (Statistical Package for Social Sciences version 25, IBM, Armonk, NY, USA). Descriptive statistics were expressed as mean±standard deviation (SD), number (n), and percentage (%). Age, testicular volume, and tissue analysis results were compared between the groups. The suitability of the data for normal distribution was evaluated with the Kolmogorov Smirnov test. The Mann-Whitney U test and independent samples t-test were used to compare parametric and non-parametric results in independent groups. The diagnostic accuracy of the results of histogram analysis was determined by receiver operating characteristic (ROC) curve analysis using the area under the curve (AUC) values with a 95% confidence interval. Statistically significant results were considered as p<0.05.

## RESULTS

A total of 100 individuals, 50 patients and 50 controls, were included in the study, and 200 testicles were examined. The average age of the patient group and the control group was 32.13±7.3 years and 31.23±7.9 years, respectively, and no statistically significant difference was detected (p=0.58) (Table 1).

**Table 1:** Age information of the patient and control groups.

Group		n	Mean	Sd	p-value
Age (years)	Patients	50	32.13	7.3	0.58^a^
	Controls	50	31.23	7.9	

n: count;

a independent samples t-test.

Sd: standard deviation.

When testicular volumes in the patient and control groups were compared, no significant difference was detected. The mean right testicular volume was found to be 14.9±2.9 mL in the patient group and 14.1±3.4 mL in the control group (p=0.45). The average left testicular volume was found to be 14.6±2.6 mL in the patient group and 14.4±3.3 mL in the control group (p=0.48).

As a result of the tissue analysis examined, a significant difference was found in six parameters. Statistically significant differences were found between the two groups in the radiomics examined in skewness of histogram, zone-size non-uniformity of gray-level size-zone matrix (GLSZM), information measure of correlation of gray-level co-occurrence matrix (GLCM), run percentage of GLRLM, high gray-level run emphasis of gray-level run-length matrix (GLRLM), and kurtosis of histogram (p=0.002, 0.002, 0.001, 0.001, 0.003, and 0.025, respectively) (Table 2). No statistically significant difference was found in other parameters.

**Table 2:** Histogram parameters and comparison of patient and control groups.

	Patient group	Control group	p	Cohen's d	95% confidence interval	
					Lower	Upper
Skewness of histogram	-0.13±0.33	0.099±0.21	0.002^a^	-0.615	-0.881	-0.310
Zone-size non-uniformity of GLSZM	0.0058±0.0022	0.0072±0.003	0.002^a^	-0.310	-0.750	-0.105
Information measure of correlation of GLCM	-0.88±0.25	-0.99±0.34	0.001^b^	-0.350	-0.712	-0.255
Run percentage of GLRLM 000Â°	0.165±0.120	0.245±0.178	0.001^a^	-0.346	-0.693	-0.002
High gray-level run emphasis of GLRLM 090Â°	3.211 (1.2–5.6)	3.36 (2.1–6.3)	0.003^b^	-0.346	-0.546	-0.256
Kurtosis of histogram	3.05 (1.99–4.12)	2.95 (1.7–3.6)	0.025^b^	-0.466	-0.746	-0.235

a Independent t-test.

b Mann-Whitney U test.

GLSZM: gray-level size-zone matrix; GLCM: gray-level co-occurrence matrix; GLRLM: gray-level run-length matrix.

## DISCUSSION

Ultrasound serves a crucial role in the diagnostic assessment of scrotal diseases. It excels in discriminating between testicular and non-testicular masses, elucidating the nature of the mass (cystic, solid, or complex). The preference for ultrasound in scrotum evaluation stems from its superior resolution, Doppler capabilities, ready availability, and the absence of ionizing radiation. The radiologist's adeptness in interpreting sonographic features associated with vascular, infectious, traumatic, and benign–malignant processes in the scrotum enables precise disease diagnosis. Noteworthy research indicates a potential correlation between acute scrotal manifestations and COVID-19^12,13^. These studies suggest that ultrasonography can reveal parenchymal findings even when acute scrotal symptoms are absent. Interestingly, findings suggestive of testicular parenchymal fibrosis were observed in the subacute and chronic phases, encompassing aspects such as reduced size, heightened echogenicity, and contour irregularities, all detectable through ultrasonography. Furthermore, several investigations propose the utility of ultrasound elastography in identifying parenchymal fibrosis^14–16^. These studies underscore the possibility of fibrosis emerging post COVID-19, potentially leading to infertility. Our study adds a novel dimension by revealing, through histogram analysis, structural disparities in the testes, indicative of parenchymal heterogeneity. This groundbreaking exploration represents the first instance of recognizing structural alterations in testicular parenchyma through ultrasound using histogram analysis.

Although histogram analysis has increased in popularity in recent years, it is still an area that is open to development. With histogram analysis, pixels and voxels in the image are examined and invisible differences can be detected. In recent years, many publications have been made on histogram analysis performed on ultrasound. In these applications, digital markers supported by computer programs called radiomics were obtained. Liang et al. showed that texture analysis would be useful in distinguishing benign–malignant thyroid nodules^12^. In their study, Xia et al. were also able to demonstrate lymph node metastasis in papillary thyroid cancer by ultrasound histogram analysis^13^. In their publication, Wang et al. demonstrated the extrathyroidal spread of papillary thyroid cancer with ultrasound texture analysis^14^. In all these studies in the literature, structural differences that are not recognizable on ultrasonography became recognizable by processing the numerical results called radiomics obtained as a result of histogram analysis. In our study, we determined the changes in the affected testicular parenchyma in accordance with the literature. Thus, together with other clinical and laboratory results, we contributed to the early detection of infertility.

With the results we obtained from histogram analysis, we have investigated which of the hundreds of parameters can best show the effect on the testicular parenchyma and we have determined six parameters. With further studies in this field, a common decision will be made on which parameters will be standardized. Knowing which parameters are more important is also necessary for artificial intelligence applications because the cutoff values in the parameters to be analyzed in artificial intelligence and deep machine learning are of great importance.

There are several limitations in our study. The first of these is the retrospective inclusion of a limited number of patients. It is difficult to prove that the volunteers in the control group did not have the disease asymptomatically, even though they did not have any active complaints at that moment and had no previous history of COVID-19 infection. Another issue is that the analysis was made only on the ultrasound images of the patients, and detailed research on infertility such as spermiogram cannot be performed. We hope that more meaningful results can be obtained in the future by taking these factors into consideration and correlating them with clinical and laboratory results. Ultrasound radiomics also suffers from the limitations of all machine learning algorithms. First, most studies have insufficient sample sizes. Adequate sample size is key to building an excellent radiomics model. If the sample size is too small, the image features extracted may not be representative and not convincing enough. It is often manifested as overfitting or overly good results on the model. Second, most ultrasound imaging groups are influenced by clinician experience. For example, the data acquisition and target segmentation processes in the workflow are uncontrollable, and these two steps are the biggest challenges concerning reproducibility in radiomics research. While certain prospective studies have assessed diagnostic models for thyroid nodules with promising results, there is a notable scarcity of prospective studies dedicated to ultrasound radiomics models^15,16^. It is imperative that future research endeavors prioritize the conduction of more prospective studies to validate the clinical feasibility of ultrasound radiomics. This imperative extends beyond thyroid applications, emphasizing the need for a broader exploration of ultrasound radiomics across various medical contexts. The scarcity of such studies underscores the importance of expanding the evidence base to ascertain the applicability and effectiveness of ultrasound radiomics as a diagnostic tool in diverse clinical scenarios.

## CONCLUSION

This study shows that texture analysis of ultrasound images of the testicles in men with COVID-19 is a potential evaluation tool. However, further research and validation studies are needed, and it is important to confirm and extend these results before moving into clinical applications. Better understanding the effects of COVID-19 on reproductive health is of great importance for managing the disease and preventing the risk of possible infertility.
